# Genomic diversity and population structure of the Leonberger dog breed

**DOI:** 10.1186/s12711-020-00581-3

**Published:** 2020-10-14

**Authors:** Anna Letko, Katie M. Minor, Vidhya Jagannathan, Franz R. Seefried, James R. Mickelson, Pieter Oliehoek, Cord Drögemüller

**Affiliations:** 1 Institute of Genetics, Vetsuisse Faculty, University of Bern, 3012 Bern, Switzerland; 2 Department of Veterinary and Biomedical Sciences, College of Veterinary Medicine, University of Minnesota, St. Paul, Minnesota 55108 USA; 3 Qualitas AG, 6300 Zug, Switzerland; 4 Dogs Global, 6871 EK Renkum, the Netherlands

## Abstract

**Background:**

Leonberger is a giant dog breed formed in the 1850s in Germany. Its post-World War II popularity has resulted in a current global population of ~ 30,000 dogs. The breed has predispositions to neurodegenerative disorders and cancer, which is likely due in large part to limited genetic diversity. However, to date there is no scientific literature on the overall demography and genomic architecture of this breed.

**Results:**

We assessed extensive pedigree records, SNP array genotype data, and whole-genome sequences (WGS) on 142,072, 1203 and 39 Leonberger dogs, respectively. Pedigree analyses identified 22 founder animals and revealed an apparent popular sire effect. The average pedigree-based inbreeding coefficient of 0.29 and average kinship of 0.31 show a dramatic loss of genetic diversity. The observed average life span decreased over time from 9.4 years in 1989 to 7.7 years in 2004. A global health survey confirmed a high prevalence of cancer and neurological disorders. Analysis of SNP-based runs of homozygosity (ROH) identified 125,653 ROH with an average length of 5.88 Mb, and confirmed an average inbreeding coefficient of 0.28. Genome-wide filtering of the WGS data revealed 28 non-protein-changing variants that were present in all Leonberger individuals and a list of 22 potentially pathogenic variants for neurological disorders of which 50% occurred only in Leonbergers and 50% occurred rarely in other breeds. Furthermore, one of the two mtDNA haplogroups detected was present in one dog only.

**Conclusions:**

The increasing size of the Leonberger population has been accompanied by a considerable loss of genetic diversity after the bottleneck that occurred in the 1940s due to the intensive use of popular sires resulting in high levels of inbreeding. This might explain the high prevalence of certain disorders; however, genomic data provide no evidence for fixed coding variants that explain these predispositions. The list of candidate causative variants for polyneuropathy needs to be further evaluated. Preserving the current genetic diversity is possible by increasing the number of individuals for breeding while restricting the number of litters per sire/dam. In addition, outcrossing would help optimize long-term genetic diversity and contribute to the sustainability and health of the population.

## Background

Leonberger is a giant dog breed that was formed around the 1850s in Germany as a watch, companion, and family dog [1]. The breed became more popular after World War II, resulting in an estimated current population of ~ 30,000 dogs [2]. Compared to other breeds, Leonberger appears to have a higher predisposition to neurodegenerative disorders and some forms of cancer such as hemangiosarcoma and osteosarcoma [3]. Recent research has shown that variants of the *ARHGEF10* [4] and *GJA9* [5] genes explain about a third of the polyneuropathy-diagnosed Leonberger cases (OMIA 001917–9615; OMIA 002119–9615), and a recessively inherited *NAPEPLD* [6] variant causes juvenile-onset leukoencephalomyelopathy (OMIA 001788–9615).

Historically, estimation of inbreeding has relied on in-depth pedigrees with inbreeding coefficients being estimated from pedigree-based relationships between ancestors (F_PED). Polymorphic microsatellite marker genotype data have been used to evaluate the genetic diversity in dogs (e.g. [7, 8]), and more recently, genome-wide single nucleotide polymorphism (SNP) genotype data have been used in several breeds [9–13]. Based on SNP data, runs of homozygosity (ROH) can be characterized, which enable quantification of the extent of inbreeding in diploid individuals, especially in the case of incomplete, unreliable, or missing pedigree information [14]. Furthermore, the length of the observed ROH segments can be used to distinguish between recent and ancient inbreeding [15, 16] and to draw conclusions about the population history of breeds [17].

For more than 10 years, livestock breeders have generated massive amounts of genotype data and implemented genomic selection schemes, but for dogs such data is mainly generated for gene-mapping purposes [18, 19]. The first use of SNP data to examine the association of measures of reproductive fitness in dogs was reported in golden retrievers and demonstrated the existence of a statistically significant negative correlation between fecundity and F_ROH [20]. In general, studies are based on SNP genotyping data of variable marker density and generally for less than 100 selected individuals per breed; e.g. Boccardo et al. [12] analysed only 34 individuals of the German shorthaired pointer breed to estimate a genomic inbreeding coefficient F_ROH of 0.17, whereas a mean inbreeding coefficient of only 0.023 was found based on genealogical information, showing the latter was incomplete.

The currently best-studied dog breed using SNP and whole-genome sequence (WGS) data to assess genetic diversity is the Norwegian lundehund, which is known to be at risk for a breed-specific multifactorial life-threatening syndrome [21]. The current lundehund population is highly inbred and optimal contribution selection alone results in no improvement due to the extremely high relatedness of the whole population [22]. SNP genotype data analyses revealed a substantially low genetic diversity in the lundehund and therefore outcrossing with closely-related breeds was recommended to rescue the endangered population [13, 22]. A successful example of replacing a pathogenic allele by outcrossing is the Dalmatian breed, which suffers from hyperuricosuria, and has an extremely high frequency of the deleterious recessive allele of the *SLC2A9* urate transporter gene. In this case, an individual from the pointer breed that was homozygous for the wild type allele of *SLC2A9* was used to produce unaffected heterozygous dogs, which were subsequently backcrossed to produce healthy individuals that are nearly indistinguishable from purebred Dalmatians [23].

WGS has enabled the generation of a large catalogue of genetic variants, which captures much of the variation that exists in modern dogs [19, 24]. A comprehensive set of variants, together with their allele and genotype frequencies within and across breeds, helps distinguish functionally relevant variants from neutral variants, and identify potential breed-specific regions of variation. These data have been shown to be highly useful for the analysis of phenotypic variation [25] and for the identification of loci that contribute to both simple and complex disease susceptibility in dogs [26, 27]. The resulting identification of causative variants contributes to maintaining the sustainability of breeds by reducing the number of inherited health problems. Data on the mitochondrial genome (mtDNA) that is also sequenced during WGS can be used as an additional indicator to assess diversity and to obtain a more comprehensive picture of the origin and history of canid populations [28–30].

To date, there is no report on the genetic characterization of the Leonberger breed using genomic data. In this paper, we used extensive pedigree data on 142,072 dogs, SNP array genotypes of 1203 dogs, and WGS data from 39 dogs to assess the genetic diversity within the current worldwide Leonberger breed population.

## Methods

### Animals

Samples from 1203 Leonberger individuals that were collected globally were used (see Additional file 1: Table S1). Genomic DNA was isolated from blood using either the Gentra PureGene blood kit (Qiagen) or the Maxwell RSC whole blood DNA kit (Promega). Thirty-nine Leonberger dogs were whole-genome sequenced either during the course of previous studies [5, 6, 31] or as unexplained cases of neurological disorders (see Additional file 1: Table S1). Pedigree records were available for 142,072 dogs through the Worldwide Independent Leonberger Database [2]. Health updates were available for 2726 dogs by owner submission through an online questionnaire.

### Pedigree analyses

We assessed extensive pedigree records including 142,072 animals with the oldest recorded date of birth in 1880 until 2016. Pedigree analyses were performed using the open source software EVA v3.0 [32] and an in-house Qualitas pedigree software that is used intensively in routine genetic evaluation analyses. Loss of genetic diversity and the potential to increase diversity were assessed by mean kinship (MK) using the tabular method as described previously by Oliehoek et al. [33]. MK of the current population was calculated for 31,832 selected dogs that were presumed to be available for breeding and alive at the time of calculation. Males born before 2009 and females born before 2011 that were assumed not to participate in breeding anymore and the known deceased dogs were excluded. Thus, the actual population size might be smaller since the current status for many animals was unknown.

### Global health survey

An online questionnaire was sent to members of various European and American Leonberger breeding clubs, and in addition, was also announced periodically via Facebook. A link to an internet-based version of the questionnaire in seven languages was provided to ensure a worldwide reach of owners and breeders [34]. The questionnaire consists of three main parts with questions on general information about the dog and owner, on signs of specific diseases such as neurological disorders and cancer, and on the overall medical history of individual dogs, including the date of death. In total, responses for 2726 dogs collected between 2013 and 2019 were analysed.

### SNP array genotyping and ROH analyses

Among the 1203 Leonberger individuals with SNP array genotype data, 308 were genotyped on the 460 k Axiom Canine Genotyping Array Set A (Thermo Fisher Scientific) and 895 on the 170 k Illumina CanineHD BeadChip (Illumina). The PLINK v1.9 software [35] was used to merge the genotype data and to perform quality control pruning and analyses of the runs of homozygosity (ROH). Merging of the different datasets was restricted to overlapping SNPs between both arrays and to SNPs with a genotype call missingness lower than 0.1. The final dataset consisted of 137,476 SNPs. In addition, only biallelic SNPs on the 38 canine autosomes were retained for the ROH analyses and SNPs that deviated from the Hardy Weinberg equilibrium (p < 0.0001) were excluded. Finally, 132,711 SNPs were available as the final dataset with one SNP per 16.52 kb. Based on a previously described method [36], we calculated that a minimum number of 70 SNPs and at least one SNP per 20 kb were required to identify a ROH and produce less than 5% randomly generated ROH. For the other parameters used to define a ROH, the default values as defined in PLINK [35] were set. An inbreeding coefficient based on ROH (F_ROH) was calculated as the total length of all ROH for one dog divided by the total length of the autosomal genome covered by SNPs. Multidimensional scaling (MDS) of pairwise genetic distances in PLINK [35] was performed to analyze the population structure. All genome positions refer to the CanFam3.1 reference sequence assembly. Figures were plotted in the R environment v3.6.0 [37] using packages CMplot v3.5.1 [38], plotrix v 3.7–6 [39], and qqman v0.1.4 [40].

### Whole-genome sequencing and variant calling

WGS data of 39 Leonberger individuals were obtained by preparing a PCR-free fragment library to generate an average 18.1 × coverage (ranging from 8.3 to 33.9 ×) (see Additional file 1: Table S1) as described previously [24]. Fastq-files were mapped to the dog reference genome assembly CanFam3.1. Variant calling of single nucleotide and small indel variants (SNVs) was performed, and their functional effects were predicted based on the NCBI annotation release 105 as described in [24]. Private variants shared by all 39 Leonberger individuals and the rare variants that were present in at least one Leonberger individual were identified by comparison with the catalogue of variants in 605 publicly available control dogs from 128 various breeds and nine wolves provided by the Dog Biomedical Variant Database Consortium [24]. A list of 113 neuropathy- and Charcot-Marie-Tooth disease-associated genes was extracted from the OMIM [41] and OMIA [42] databases (see Additional file 2: Table S2). These genes were used to filter all 653 available genomes for enriched variants in the sequenced Leonberger dogs and the alternative allele frequency was calculated to detect uncommon variants associated with disease and possibly shared by other breeds related to the Leonberger. Several *in silico* prediction tools, PROVEAN [43], MutPred2 [44], MutPred-Indel [45], MutPred-LOF [46], and PredictSNP [47], were used to predict the biological consequences of the discovered variants on the corresponding proteins. Variability of the mitochondrial genome was explored to determine the diversity in the haplogroups of the studied dogs based on the nomenclature described by both Pereira et al*.* [48] and Duleba et al*.* [29]. Integrative Genomics Viewer was used to visually inspect and confirm the detected SNVs [49].

## Results

### Pedigree analyses

Pedigree information from 142,072 Leonberger individuals was available with a pedigree completeness index across five generations higher than 99% for the animals from the latest cohorts and exceeded 80% in 1935. More than 4000 dogs were born worldwide each year since 2000 (see Additional file 3: Figure S1). We observed an average litter size across cohorts of 6.5 puppies and a constant generation interval of 4 years. In summary, 22 founder animals were identified (10 males, 12 females) among which three (2 males and 1 female) contributed almost 46% to the last cohort (year of birth 2016). The Leonberger breed underwent a severe bottleneck during the 1940s with only 17 inbred dogs (F_PED ranging from 0.14 to 0.35) registered in 1946 (see Additional file 3: Figure S1).

**Fig. 1:**
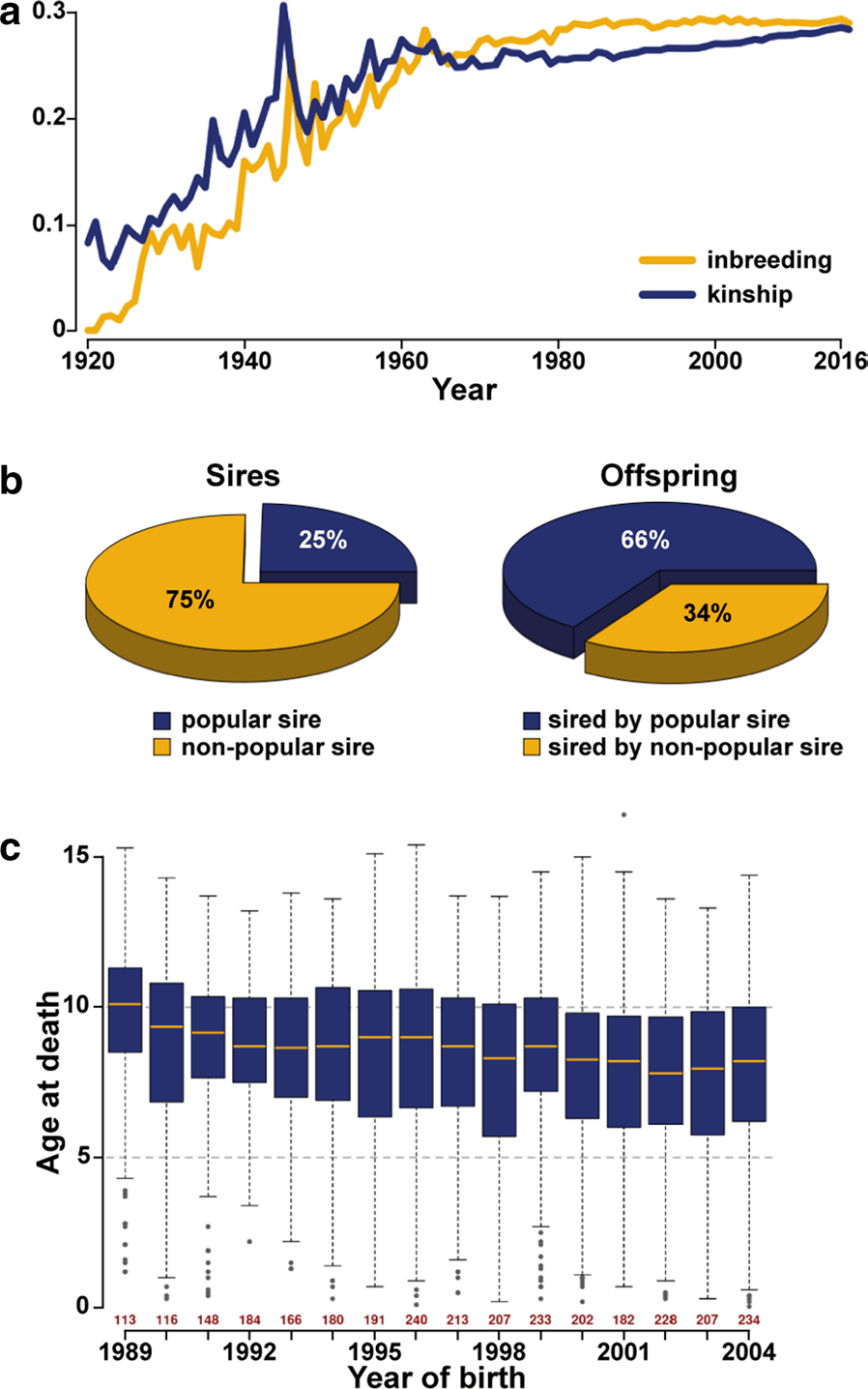
Graphical representation of the pedigree analyses of Leonberger dogs. **a** Average pedigree-based inbreeding coefficient and kinship estimated per year from 1920 to 2016. **b** Pie charts of the proportion of sires used in breeding clearly showing the popular sire syndrome. Popular sires are males that produced at least 33 puppies and account for 25% of all breeding males. These sires are responsible for 66% of all offspring. **c** Boxplot of age at death per year of birth showing the life expectancy of Leonberger dogs over years 1989–2004. The exact number of records available per year is indicated in red above each year

The average pedigree-based inbreeding coefficient (F_PED) and the average kinship showed a steady increase over the years analysed, with an estimated F_PED of 0.29 in the last cohort (year of birth 2016) (Fig. 1a). A popular sire was defined as a male dog that sired at least 33 puppies, corresponding to five litters based on the observed average litter size. Information on the number of offspring was available for 5456 sires born between 1894 and 2016. A popular sire effect was evident since a quarter of all sires produced two thirds of all offspring (Fig. 1b). In addition, although the top breeding male who sired 434 dogs was born in 1985, the second and third top breeding males who sired 394 and 337 registered puppies, respectively, were both born in 2003. This trend is visible throughout the breeding history of Leonbergers (see Additional file 3: Figure S1): the top 27 sires, which were born between 1976 and 2010, produced more than 200 puppies each (corresponding to at least 30 litters per sire). In addition, the date of death was known for 4783 dogs (2453 females and 2330 males) born between 1959 and 2019. Life expectancy was further evaluated for the period between 1989 and 2004 for which more than 100 records were available and we assumed that there were no more living dogs. This cohort included 3044 dogs (1460 males and 1584 females) and revealed a mean of 8.2 (median 8.5) with the male longevity (mean = 7.9) being on average lower than the female longevity (mean = 8.5). In addition, a slow continuous decrease across time was observed, from an average of 9.4 years in 1989 to 7.7 years in 2004 (median: 10.1 to 8.2 years) (Fig. 1c).

Mean kinship was analysed on a set of 31,832 Leonberger dogs that were presumed to be available for breeding and the resulting average kinship was 0.31. The difference between minimum (0.29) and maximum (0.33) values of MK was small, and no families that would be highly unrelated to the overall population were found. Three groups color-coded as green (MK < 0.31), yellow (MK = [0.31–0.32]), and orange (MK > 0.32) were created for better visualization. The proportion of dogs with higher or lower MK differed between countries (see Additional file 4: Figure S2). The color-coded MK coefficients have been incorporated into the Worldwide Independent Leonberger Database [2].

### Leonberger dogs are at risk for several health disorders

We collected medical data throughout the life of 2726 Leonberger dogs from owner-submitted health updates of their dogs. Of those 2726 Leonberger dogs, 1334 (48.9%) suffered from at least one health condition (see Additional file 5: Table S3). However, individual dogs (n = 544) often suffered from multiple disorders. In total, 586 (21.5%) dogs have reported tumor or cancer, with osteosarcoma (42.5%) and hemangiosarcoma (22.5%) being the two most frequent types. Other frequently seen groups of health issues included orthopaedic (15.8%), neurological (14.8%), endocrine (5.6%), digestive (4.2%), and cardiac (4.1%) problems. Respectively, the most frequent specific disorder in each system category was arthritis (222 cases), polyneuropathy (362 cases), hypothyroidism (142 cases), gastric torsion (54 cases), and dilated cardiomyopathy (45 cases). All reported disorders and the number of dogs suffering from them are listed in Additional file 5: Table S3. Most of the polyneuropathy-affected dogs (71.0%) showed both breathing and gait abnormalities, whereas for 14.6% only laryngeal paralysis or breathing problems, and for 14.4% only gait abnormalities were reported (see Additional file 5: Table S3).

### Multidimensional scaling analysis of worldwide Leonberger population structure

**Fig. 2:**
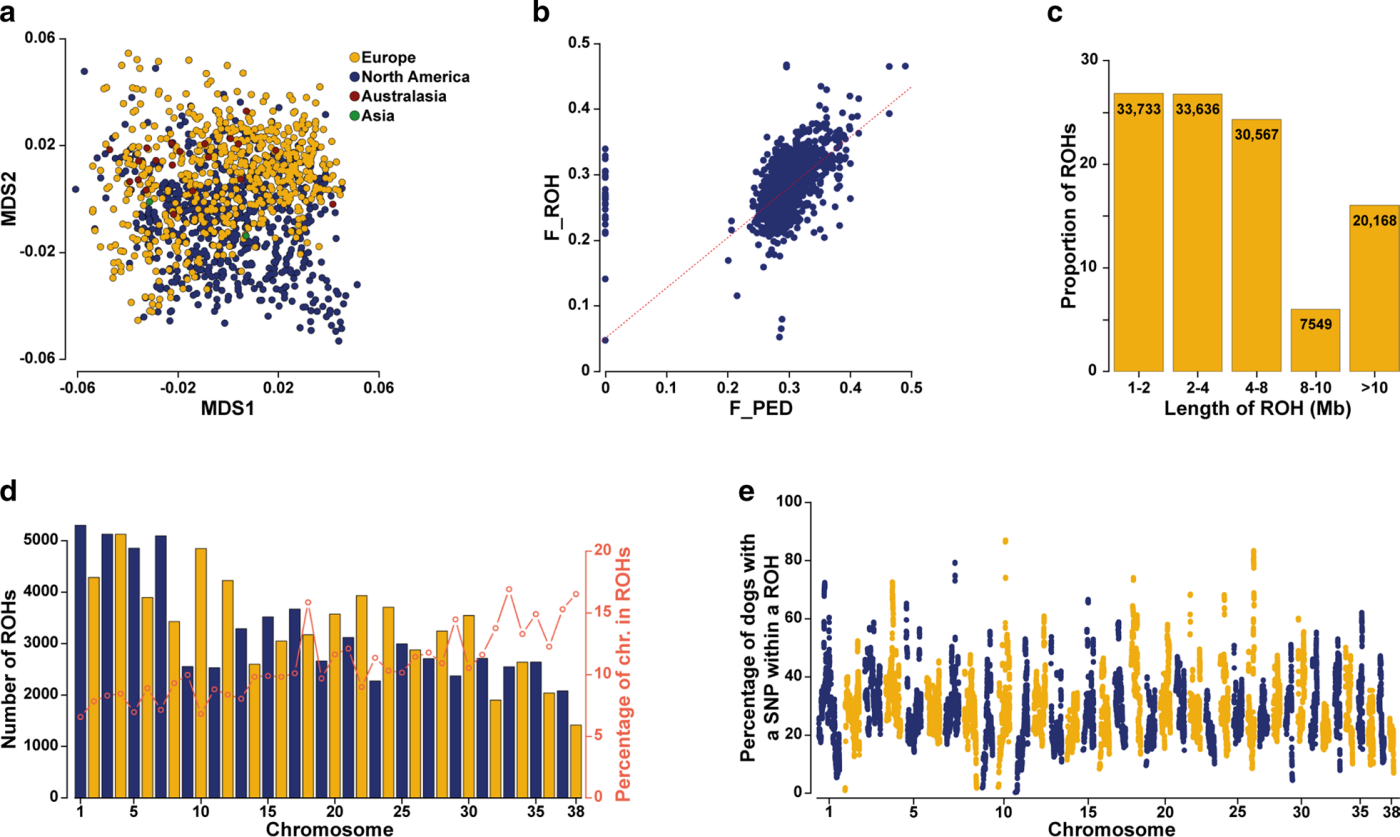
Graphical representation of the genomic analyses of Leonberger dogs. **a** MDS plot of 1203 dogs showing no obvious subpopulations regardless of the geographical region of residence. **b** Correlation between inbreeding coefficients estimated from the pedigree data (F_PED) and the ROH analysis (F_ROH). Note that the dogs with unknown pedigrees (F_PED = 0) on the left side mostly show the same level of inbreeding as the dogs with known registered records. **c** Frequencies and counts of the different classes of ROH based on their length indicating both old and recent inbreeding. **d** Number of detected ROH per autosome (left y axis) and the proportion of chromosomes in a ROH (right y axis). **e** Manhattan plot showing the proportion of dogs sharing a SNP within a ROH across the 38 autosomes. Most dogs share a region on chromosomes 10 and 26

The 1203 dogs genotyped on SNP arrays represented well the Leonberger population because they were sampled throughout time and from various countries (see Additional file 1: Table S1). Subpopulations were expected due to the large geographic distances between the dogs: 579 samples (48.1%) came from the USA and Canada, 602 samples (50.1%) came from European countries, and 22 samples (1.8%) came from Australia, New Zealand, or Japan. However, multidimensional scaling (MDS) of pairwise genetic distances revealed no obvious clustering (Fig. 2a) and (see Additional file 6: Figure S3), although a small group of North American dogs did not overlap completely with the rest of the population. In light of Additional file 6: Figure S3b, the non-overlapping dogs were mostly born before 2000, whereas the more recently born dogs seem to be closer to the European population. In addition, the obvious difference in the number of genotyped dogs born in or before 2000 from North America (199 of 579) and the number of those from Europe (88 of 602) has to be taken into account, since this disproportion in sampling might contribute to the incomplete overlap.

### Analysis of runs of homozygosity

The ROH-based genomic inbreeding coefficient (F_ROH) was on average 0.28 (ranging from 0.05 to 0.47). In addition, the comparison between F_PED and F_ROH revealed a few dogs with obviously incorrect pedigree records and enabled the estimation of inbreeding in individuals with missing or unknown pedigrees (Fig. 2b) and (see Additional file 1: Table S1). In total, 125,653 ROH with an average length of 5.88 Mb (ranging from 1.00 to 90.17) were identified. ROH could be divided into five groups based on their size (Fig. 2c). Most of the detected ROH (53.6%) were shorter than 4 Mb and 16.1% were longer than 10 Mb (Fig. 2c). The number of ROH per chromosome ranged from 1419 on chromosome 38 to 5303 on chromosome 1; the proportion of overall chromosomal regions involved in homozygous regions relative to chromosomal size was highest for chromosome 33 (16.9%) and lowest for chromosome 1 (6.6%) (Fig. 2d). On average, 104.4 ROH were detected per dog with a range from 37 to 177. The total length of ROH across the genome per individual was on average 614 Mb and ranged from 104.6 to 1028.2 Mb. The proportion of dogs with a SNP within a ROH varied across the 38 autosomes as shown in Figure S4 [see Additional file 7: Figure S4]. For chromosome 10 (chr10: 44,529,255–44,831,343), 1047 of the 1203 dogs (87.0%) shared a ROH across ~ 302 kb. Only one protein-coding gene is annotated in this region, *TMEM131*, which encodes a transmembrane protein not known to be associated with any disease. This protein is assumed to play a role in the development and differentiation of T cells and in collagen recruitment and secretion [50, 51]. A ~ 236 kb region (chr26:35,428,129–35,663,964) at the end of chromosome 26 was shared by 1000 (83.1%) of the 1203 analysed dogs (Fig. 2e). Only two pseudogenes and one uncharacterized ncRNA are annotated in this region. BLAST-based comparisons of these loci revealed no significant sequence similarity to human genes.

### Whole-genome sequence analysis identified no protein-changing variants fixed in Leonberger dogs

The WGS data of 39 Leonberger dogs including 38 individuals with variable neurological signs (see Additional file 1: Table S1) were compared to 614 publicly available canid WGS [24]. These sequenced Leonbergers included four individuals that were sequenced to identify the pathogenic variants in the *GJA9* and *NAPEPLD* genes described previously [5, 6]. First, we searched for Leonberger breed-specific variants that were absent from the control genomes and identified only 27 non-coding and one coding private variants that were shared by all 39 Leonberger dogs at least in the heterozygous state (see Additional file 8: Table S4). Nine of these variants were located close to the detected ROH intergenic region on chromosome 26. The only coding variant was a synonymous SNV on chromosome 1 in the *ZNF510* gene that encodes a relatively little-studied zinc finger protein.

### Leonberger-specific protein-changing variants as new candidates for neuronal disorders

Next, we filtered for rare variants (i.e. alleles that occurred in the genome of at least one Leonberger dog and absent from the 614 control genomes), which led to the discovery of 62,531 SNVs, of which 1379 were predicted to be coding (see Additional file 9: Table S5). Of those, 855 were predicted to be protein-changing with the alternative allele frequency ranging from 0.01 to 0.63 (see Additional file 9: Table S5). Considering the genes that are associated with inherited neuronal disorders in other species, we found 11 potentially pathogenic variants (Table 1). This short list included two previously known pathogenic variants such as the leukoencephalomyelopathy-associated missense variant in *NAPEPLD* [6] at a frequency of 0.12 and the polyneuropathy-associated frameshift variant in *GJA9* [5] at a frequency of 0.05. In addition to a single missense variant predicted to be neutral, eight of the other nine variants were predicted as probably deleterious including two missense, two frameshift, two in-frame-insertions, one in-frame-deletion, and one nonsense variant (Table 1) and (see Additional file 9: Table S5 and Additional file 10: Table S6).

**Table 1:** Rare protein-changing private variants in Leonberger dogs

Gene	OMIM/ OMIA number	Variant designation^a^			Alternative allele frequency
		Genomic position	Coding DNA change	Protein change	
*ATXN7*	607640	chr20:27,234,549	c.2285 T > C	p.Val762Ala	0.1154
*CEP55*	610000	chr28:7,777,489	c.1334A > C	p.Tyr446Ser	0.0256
*CNTF*	118945	chr18:37,758,771	c.401_402insA	p.Asn137fs	0.0128
*ELOVL4*	605512	chr12:40,850,011	c.424_433delGGAGCACAGC	p.Gly142fs	0.0128
*FDX1L*	614585	chr20:50,798,661	c.124_125insGGCCGCCATCACCACGGCGGTGAGCACCGCCGCCAGCAGCACCAGCCCGTCAGCGTTGAGCCG	p.Thr41_Ala42insGlyProProSerProArgArgTer	0.0128
*GJA9*^b^	611923/2119–9615	chr15:3,863,519	c.1108_1109delGA	p.Glu370fs	0.0513
*MCM3AP*	603294	chr31:39,553,337	c.1785_1793delCTCTGAAGG	p.Ser596_Gly598del	0.0128
*NAPEPLD*^c^	612334/1788–9615	chr18:16,987,520	c.559G > C	p.Ala187Pro	0.1154
*PLEKHG5*	611101	chr5:60,325,903	c.1585C > T	p.Gln529*	0.0128
*SPTBN4*	606214/2232–9823	chr1:113,215,064	c.1247_1248insGGTAGCCCATGCGGT	p.Ala416_Ala417insValAlaHisAlaVal	0.0128
*SYNE1*	608441	chr1:42,549,994	c.17359C > T	p.Arg5788Trp	0.0256

^a^Additional details including the protein prediction effects are described in Additional file 10 Table S6. All positions refer to the CanFam3.1 reference sequence assembly

^b^Previously described polyneuropathy-associated variant [5]

^c^Previously described leukoencephalomyelopathy-associated variant [6]

### Protein-changing variants in candidate genes for neuronal disorders enriched in Leonberger dogs

Because 33 of the 39 sequenced Leonberger individuals suffered from an unexplained form of polyneuropathy (see Additional file 1: Table S1), additional filtering was done for variants that were present in 113 putative candidate genes (see Additional file 2: Table S2) and could be involved in the development of the disease. Focusing on coding variants that occurred in at least one sequenced individual resulted in 649 variants that affected 100 genes, among which 232 were predicted to be protein-changing (see Additional file 11: Table S7). All these variants were also present at variable frequencies in other dog breeds and wolves.

To search for variants that are enriched in Leonberger dogs, we considered those with an alternative allele frequency higher than 0% and lower than 10% in the 614 control canids and that were more than twice as common in the Leonberger dogs as in the controls. This approach yielded a short list of 22 SNVs that affect 17 genes including 21 missense and one in-frame-deletion variant (Table 2). In addition to the 11 variants predicted to be neutral, 11 other variants were predicted as most likely deleterious and represented potentially pathogenic variants causing polyneuropathy (Table 2) and (see Additional file 10: Table S6). Interestingly, a new potentially deleterious missense variant was found in a different codon in the *GJA9* gene in which a variant has already been reported to be associated with polyneuropathy in Leonbergers [5]. This newly discovered *GJA9* variant occurred at a quite high frequency (0.55) within the sequenced cohort of Leonberger dogs but was also comparatively common (frequency of 0.09) among the control dogs from 35 breeds (see Additional file 9: Table S5). The other variants occurred at different frequencies in several breeds (see Additional file 9: Table S5). Only one missense variant in the *DST* gene was quite rare, i.e. it was present in the heterozygous state in only one Howavart dog (see Additional file 9: Table S5).

**Table 2:** Protein-changing variants in polyneuropathy-associated candidate genes enriched in Leonberger dogs compared to 614 controls from various breeds

Gene	OMIM/ OMIA number	Variant designation^a^			Alternative allele frequency	
		Genomic position	Coding DNA change	Protein change	Leonbergers	Controls
*CNTNAP1*	602346	chr9:20,294,320	c.3863G > C	p.Arg1288Pro	0.3974	0.0366
*CNTNAP1*	602346	chr9:20,298,261	c.2585G > A	p.Gly862Glu	0.1538	0.0067
*DHTKD1*	614984	chr2:24,316,951	c.1820C > T	p.Ala607Val	0.0897	0.0049
*DIAPH3*	614567	chr22:15,880,854	c.2000G > A	p.Cys667Tyr	0.1538	0.0689
*DST*	113810	chr12:23,771,235	c.18224C > T	p.Thr6075Met	0.0769	0.0008
*DST*	113810	chr12:23,782,332	c.17800C > T	p.Arg5934Trp	0.4359	0.0330
*DST*	113810	chr12:23,846,624	c.10661C > T	p.Ser3554Leu	0.0769	0.0369
*DYNC1H1*	600112	chr8:70,064,306	c.13757C > T	p.Pro4586Leu	0.1282	0.0051
*GARS*	600287	chr14:43,322,005	c.622G > A	p.Val208Ile	0.1154	0.0025
*GJA9*	611923/2119–9615	chr15:3,862,761	c.344G > C	p.Arg115Thr	0.5513	0.0868
*JPH1*	605266	chr29:22,710,800	c.1531A > G	p.Ile511Val	0.1923	0.0299
*LOC477508*	602072	chr26:16,348,822	c.298G > A	p.Val100Met	0.1795	0.0066
*MME*	120520	chr23:49,045,461	c.1700A > T	p.Gln567Leu	0.0769	0.0157
*NEFH*	162230	chr26:22,729,485	c.1046G > A	p.Arg349His	0.2821	0.0116
*NEFH*	162230	chr26:22,732,974	c.1901_1924delTGAAGGAGGAGGCCAAGTCCCCAG	p.Val640_Pro647del	0.3974	0.0849
*NEFH*	162230	chr26:22,733,415	c.2326C > G	p.Pro768Ala	0.0513	0.0059
*OTOF*	603681	chr17:20,534,866	c.3451G > A	p.Ala1151Thr	0.7051	0.0094
*PDXK*	179020	chr31:37,707,445	c.774G > C	p.Arg258Ser	0.1923	0.0898
*PRX*	605725	chr1:113,290,407	c.784G > A	p.Ala262Thr	0.1282	0.0257
*SBF2*	607697	chr21:33,036,988	c.4114C > T	p.Pro1372Ser	0.1667	0.0361
*SLC12A6*	604878	chr30:853,540	c.2498C > T	p.Ala833Val	0.0513	0.0250
*WNK1*	605232	chr27:42,911,057	c.7448C > G	p.Thr2483Arg	0.0128	0.0058

^a^Additional details including the protein prediction effects are described in Additional file 10: Table S6. All positions refer to the CanFam3.1 reference sequence assembly

### Mitochondrial genome diversity is limited in Leonberger dogs

Finally, we evaluated the diversity of the mitochondrial genome of the 39 studied Leonberger dogs and detected only two haplotypes. Based on the nomenclature standard using only the mtDNA D-loop [48], 38 dogs showed the A2 haplotype and one dog the A17 haplotype. The same results were obtained when using the complete mitogenome database standardized haplogroup nomenclature [29] with 38 dogs belonging to the A1b2a1a1 haplogroup and the one dog to the A1b1a1a haplogroup (see Additional file 12: Table S8). In addition, 10 SNVs were detected in the coding part of the mtDNA, among which seven were private, occurring rarely in individual Leonbergers and were not found in the 614 controls (see Additional file 12: Table S8). This list included two variants that affect tRNA genes and a single missense variant in the *ND2* gene observed in one dog only (Table 3).

**Table 3:** Private variants in mitochondrial DNA of Leonberger dogs

Gene	Variant designation^a^	Proportion of variant^b^	OMIM/OMIA number	Type
*tRNA-Phe*	m.49A > G	96%	590070	Unknown
*tRNA-Val*	m.1069 T > C	99%	590105	Unknown
*ND2*	m.4764 T > C; p.Ile284Thr	100%	516001	Missense
*COX1*	m.5681A > G	66%, 89%	516030	Synonymous
*ND4*	m.11211C > T	72%, 66%	516003	Synonymous
*ND5*	m.13118 T > C	62%	516005	Synonymous
*ND5*	m.13544A > G	100%, 100%, 100%, 100%	516005	Synonymous

^a^All positions refer to the CanFam3.1 reference sequence assembly

^b^Percentage of variant represents heteroplasmy, 100% represents homoplasmy, for each dog that carries the variant calculated from the coverage of the affected mtDNA region

## Discussion

This is the first study to comprehensively describe the genomic diversity and population structure of the Leonberger breed by exploring the distribution of ROH and the SNV based on SNP array and WGS data. In addition, the genealogical pedigree information was used to compare the estimated inbreeding coefficients and mean kinship in the current population. The new genomic regions reported here might underlie breed-specific characteristics and provide information about the genetic structure of the breed, such as selection pressure on specific traits, inbreeding levels, or genetic bottlenecks.

Pedigree analyses based on extensive data revealed average litter size and life expectancy for the Leonberger dogs included in our study that are in agreement with previously reported results [52]. Long ROH segments (~ 10 Mb) occur as a result of recent inbreeding, whereas short ROH (< 2 Mb) indicate genomic regions that are identical by descent from older ancestors [12]. In the Leonberger dogs studied here, the 1- to 2-Mb segments account for approximately 25% of the detected ROH and those that are longer than 8 Mb account for about 20% (Fig. 2c), which indicates both old and recent inbreeding. This is in accordance with the known history of the breed and the observed current breeding practices. Smaller-sized ROH are challenging to detect because of the limited number of markers and their varying density across the genome. For example, the highly shared ROH on chromosome 26 for which 83% of the dogs were homozygous was confirmed by the identification of nine variants that were shared by all 39 sequenced dogs.

The pedigree analysis revealed the occurrence of a clear bottleneck during the 1940s and the extensive use of popular sires and line-breeding that resulted in the recent high kinship. A limited number of ancestors with high genetic contribution that had a big impact on within-breed relatedness, resulted in inbreeding due to this relatedness, and in turn inbreeding depression. It is likely that some disease-causing genetic variants that these ancestors carried are now spread throughout the entire population. Moreover, the MDS analysis did not show any clear differentiation based on geographical or purpose lineages in the Leonberger population, which contrasts with previous results in other breeds, e.g. the English greyhounds and Labrador retrievers that cluster based on specific working and show lineages or the Italian greyhounds and Shetland sheepdogs that form discrete clusters separating the European and North American populations [53].

Our worldwide health survey of almost 3000 Leonberger individuals confirmed a high prevalence of cancer, particularly osteosarcoma and hemangiosarcoma, neuromuscular disorders, and hypothyroidism. In other dog breeds, genetic association studies that aimed at identifying genetic risk factors [27] have revealed a strong association with specific genome regions, e.g. for osteosarcoma [54, 55]. Similar studies could be performed in the Leonberger breed to unravel such associations and identify markers for selection against the elevated risk of developing certain forms of cancer. In addition, hypothyroidism was recorded quite frequently in our survey, confirming earlier reports for this breed [56, 57]. However, diseases such as arrhythmia leading to sudden cardiac death [58] that has been reported to be more prevalent in Finnish Leonbergers occurred only sporadically in our survey. Besides the orthopaedic disorders, which are known to be polygenic, the second most prevalent disease category that was reported in our survey was neurological disorders, with predominantly different forms of polyneuropathy as reported earlier [59]. A recent survey conducted by the Leonberger Health Foundation International based on more than 1000 dogs from 24 countries also highlighted the high prevalence of certain forms of cancer such as osteosarcoma and hemangiosarcoma, as well as that of neurological disorders, such as laryngeal paralysis and other forms of polyneuropathy [60]. Although these two surveys were not truly comprehensive and possibly biased towards selected conditions, they were performed independently at different times, and therefore we considered that they provided reasonable estimates of the true frequencies.

We sequenced the genomes of more than 30 polyneuropathy-affected dogs with the aim of unravelling additional disease-causing variants. The WGS data provided evidence for both breed-specific and enriched variants in the genomes of polyneuropathy-affected Leonbergers at different frequencies. The short list of 21 potentially pathogenic variants in candidate genes for neurological disorders includes two previously reported variants: one in *GJA9* that causes polyneuropathy [5] and one in *NAPEPLD* that causes leukoencephalomyelopathy [6]; these findings confirm the potential usefulness of the approach chosen. The newly discovered variants are well-known candidates for neurological disorders in various species. Future genotyping of the 19 newly identified potentially deleterious protein-changing variants in cohorts of well-phenotyped Leonberger dogs is needed to establish association of these variants with diseases. The second potentially pathogenic missense variant in *GJA9* is compelling since it is also present at a lower frequency in dogs of unrelated breeds. Two of the three mtDNA SNV that affect tRNA genes will also be interesting to follow-up because a variant in the *tRNA-Tyr* gene causes a familial form of sensory ataxic neuropathy in golden retrievers [61]. Since, to date, we have been unable to identify additional loci that are associated with polyneuropathy disorders inherited in a Mendelian fashion by genome-wide association study, we believe that a subset of these disorders could have a complex genetic nature and that the individual’s likelihood of developing the disorder depends on a combination of multiple alleles at multiple loci in addition to environmental factors. Future investigation of the presence of large structural variations throughout the Leonberger genome would also be informative when an improved canine reference assembly, an improved annotation, and long-read sequence data become available. Earlier studies proposed the presence of a sex bias, since more male Leonbergers are affected with polyneuropathy than female Leonbergers [62], but to date no major risk factor has been identified on the X chromosome.

## Conclusions

Considerable genetic diversity has been lost in the Leonberger breed due to a bottleneck that occurred during the last century and to an increasing population size thereafter. This situation appears to be due primarily to the use of popular sires resulting in high levels of inbreeding, which has also facilitated the spread of undesirable genetic traits within the gene pool. Maintaining the current level of genetic diversity will only be possible through informed selection decisions, especially by including more dogs in breeding programs, avoiding repeated matings and the use of popular sires, and minimizing coancestry among the selected parents. The established mean kinship groups may also help breeders to select the most suitable mating pairs in addition to the already used pedigree-based inbreeding coefficients. In addition, careful outcrossing might help optimize long-term genetic diversity, increase heterozygosity, and reduce the frequency of disease-causing alleles in order to lower the incidence of various health problems. The list of putative pathogenic variants in candidate genes for neurologic and neuromuscular diseases provided in this paper might enable the identification of new disease-causing mutations in the future. Our results also illustrate the most likely heterogeneous genetic architecture of this group of diseases that was thought to be Mendelian, but resembles more and more a polygenic complex disorder.

## Supplementary information

**Additional file 1: Table S1**. List of the 1203 dogs analysed in this study with available genomic information. The table includes supplementary descriptive details of the studied Leonberger dogs including accession numbers and known health history of the WGSed dogs.

**Additional file 2: Table S2**. List of 113 selected genes used for filtering variants in the 653 available WGS data. Putative candidate genes and their phenotype association in humans or animals based on OMIM [41] and OMIA [42] databases showing the number of variants detected in each of these genes in the Leonberger genomes.

**Additional file 3: Figure S1**. Population size of the Leonberger breed between the years 1920–2016. The blue, yellow, and green lines represent the number of breeding males, breeding females, and puppies they produced per year, respectively. The red dashed line represents the number of expected parent pairs for the given number of born puppies per year, assuming the average litter size of 6.5 puppies. Panel (**a**) shows the increasing population size from 1920 to 1978 and the apparent bottleneck around 1946. In the 1970s, the number of dogs born started to increase rapidly whereas the number of breeding males used started to decrease. Panel (**b**) shows the continuously increasing population from 1979 to 2016. Note that the number of dams is more or less as expected but the number of sires constantly decreases to about half of that of the dams in recent years, illustrating the popular sire syndrome.

**Additional file 4: Figure S2**. Mean kinship of the estimated current population of 31,832 Leonberger dogs per country. The proportion of dogs belonging to the three groups that indicate the increasing relatedness to the whole population, is shown as green (MK < 0.31), yellow (MK = [0.31–0.32]), and orange (MK > 0.32) by country. The total number of dogs recorded in each country is shown in red at the top of the corresponding columns.

**Additional file 5: Table S3**. Distribution of disease phenotypes reported in 2726 Leonberger dogs. The table shows the number of dogs for which the owners reported particular disorders. Each more general group is divided in specific subgroups. Note that many dogs have multiple health issues and therefore the sum of the individual counts will be larger than the total of 1334 dogs with at least one health condition reported.

**Additional file 6: Figure S3**. MDS plots of the 1203 Leonberger dogs highlighted by different groups. Description: Panel (**a**) shows the distribution of the dogs by country. Panel (**b**) shows the dogs divided into five groups by their year of birth. This is also reflected in panel (**c**), where the dogs are coded by their calculated MK coefficient, which was not determined for the older dogs.

**Additional file 7: Figure S4**. Proportion of dogs with a SNP within a ROH on each of the 38 autosomes. Individual Manhattan plots showing the percentage of 1203 dogs genotyped on the SNP array that share a SNP within a ROH for each canine autosome.

**Additional file 8: Table S4**. List of private whole-genome sequence variants in 39 Leonberger dogs. Breed-specific SNVs present at least in the heterozygous state in all Leonberger dogs and absent from the 614 control genomes.

**Additional file 9: Table S5.** List of rare whole-genome sequence variants in 39 Leonberger dogs. Breed-specific SNVs present in at least one Leonberger dog and absent from the 614 control genomes.

**Additional file 10: Table S6**. List of the potentially pathogenic variants for neurological disorders detected in 39 Leonberger dogs including the predictions of the effect of the variants on the proteins. The table includes detailed description and predictions of the biological consequences of the discovered variants on the corresponding proteins using different in silico prediction tools: PROVEAN [43], the MutPred suite (includes MutPred2 [44], MutPred-Indel [45], MutPred-LOF [46]), and PredictSNP [47].

**Additional file 11: Table S7**. List of whole-genome sequence variants present in at least one Leonberger dog in the 113 selected genes showing genotypes of all 653 available genomes. SNVs present in polyneuropathy- and Charcot-Marie-Tooth disease-associated candidate genes (listed in Additional file 2: Table S2) and their alternative allele frequency in the Leonberger dogs and 128 other dog breeds and wolves.

**Additional file 12: Table S8**. Diversity of the mitochondrial genome of 39 Leonberger dogs. The table shows the low diversity of Leonberger mtDNA based on two nomenclature standards [46, 48]: most dogs belong to the A1b2a1a1 or A2 haplogroup whereas only one dog belongs to the A1b1a1a or A17 haplogroup. In addition, seven private SNVs detected in the mtDNA of the Leonberger dogs are also highlighted.

## Data Availability

The WGS dataset generated and analysed during the current study is available in the European Nucleotide Archive (https://www.ebi.ac.uk/ena). The 39 Leonberger dog genomes have been made freely available under study accession number PRJEB16012 and all sample accession numbers are listed in Table S1 (see Additional file 1: Table S1). The SNP genotyping data is available on reasonable request.
