# Correction to: Genomic diversity and population structure of the Leonberger dog breed

**DOI:** 10.1186/s12711-020-00590-2

**Published:** 2020-11-18

**Authors:** Anna Letko, Katie M. Minor, Vidhya Jagannathan, Franz R. Seefried, James R. Mickelson, Pieter Oliehoek, Cord Drögemüller

**Affiliations:** 1 Institute of Genetics, Vetsuisse Faculty, University of Bern, 3012 Bern, Switzerland; 2 Department of Veterinary and Biomedical Sciences, College of Veterinary Medicine, University of Minnesota, St. Paul, MN 55108 USA; 3 Qualitas AG, 6300 Zug, Switzerland; 4 Dogs Global, 6871 EK Renkum, The Netherlands

## Correction to: Genet Sel Evol (2020) 52:61 10.1186/s12711-020-00581-3

After publication of the original article [1], the authors noticed a typesetting error in the hyper-linking of the additional files. The additional files have been relinked and can be accessed via the original article.

## References

[1] Letko A, Minor KM, Jagannathan V, Seefried FR, Mickelson JR, Oliehoek P, Drögemüller C (2020). Genomic diversity and population structure of the Leonberger dog breed. Genet Sel Evol.

