# Supplementary figures and images for: Genomic diversity and population structure of the Leonberger dog breed
Source: Genet Sel Evol. 2020 Oct 14;52:61. doi: 10.1186/s12711-020-00581-3 (PMC7557023; doi:10.1186/s12711-020-00581-3)

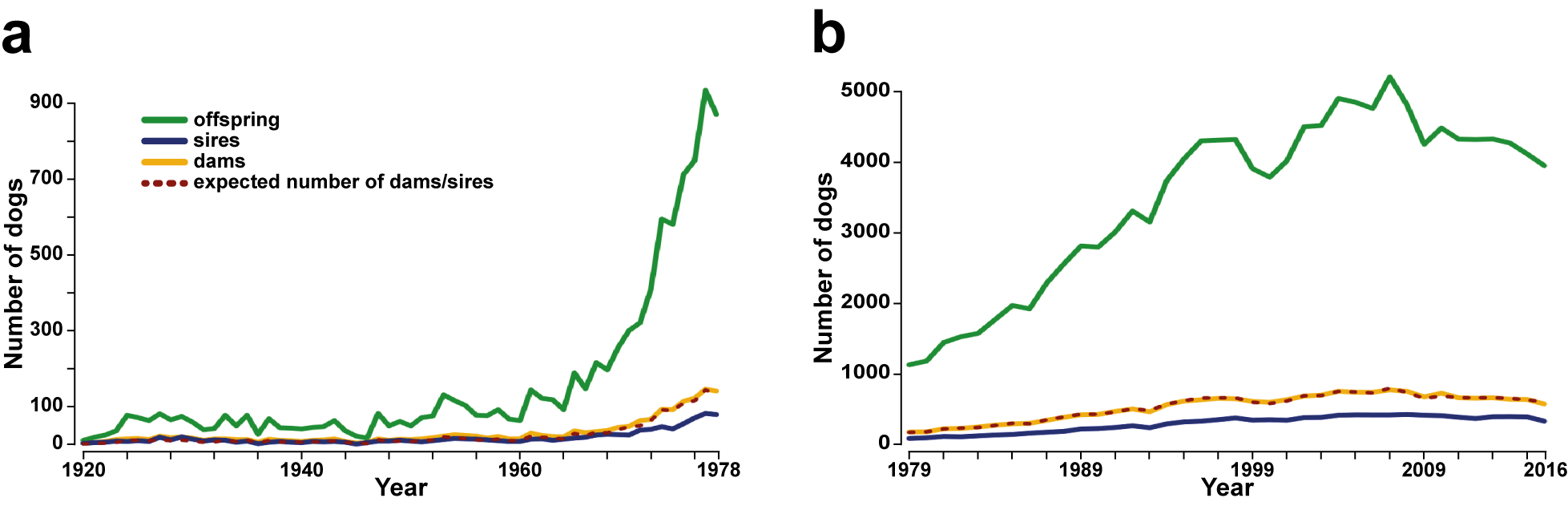

Supplement: Supplementary file 3 — Additional file 3: Figure S1. Population size of the Leonberger breed between the years 1920–2016. The blue, yellow, and green lines represent the number of breeding males, breeding females, and puppies they produced per year, respectively. The red dashed line represents the number of expected parent pairs for the given number of born puppies per year, assuming the average litter size of 6.5 puppies. Panel (a) shows the increasing population size from 1920 to 1978 and the apparent bottleneck around 1946. In the 1970s, the number of dogs born started to increase rapidly whereas the number of breeding males used started to decrease. Panel (b) shows the continuously increasing population from 1979 to 2016. Note that the number of dams is more or less as expected but the number of sires constantly decreases to about half of that of the dams in recent years, illustrating the popular sire syndrome. [file 12711_2020_581_MOESM3_ESM.tif]

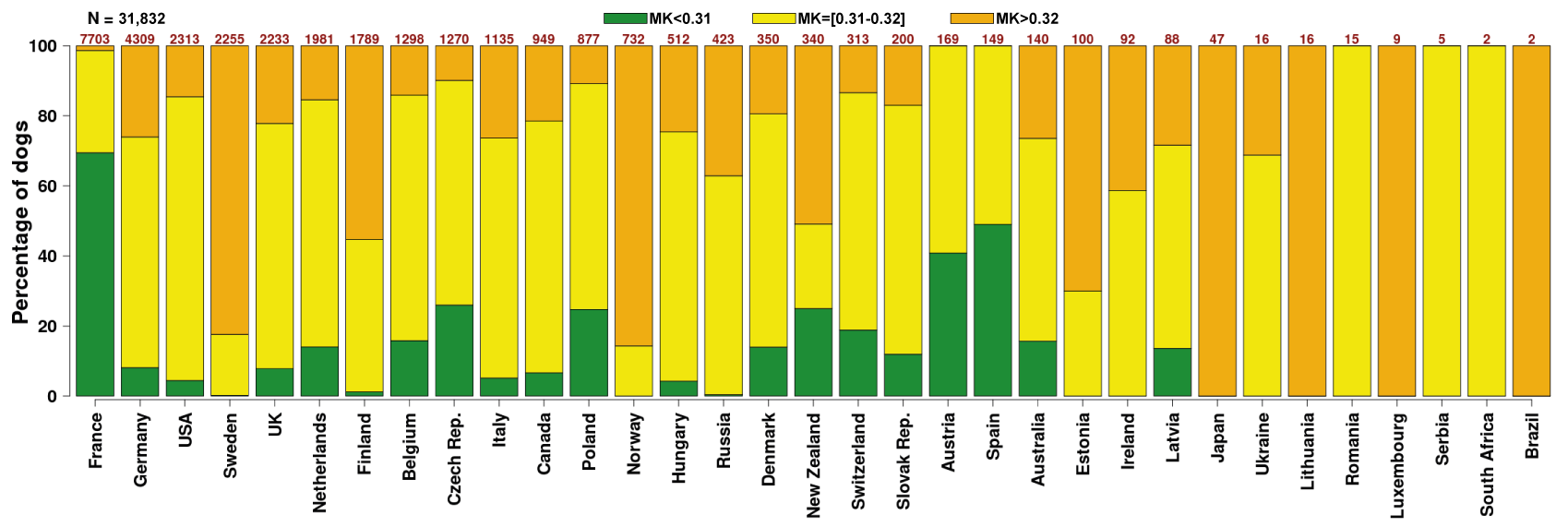

Supplement: Supplementary file 4 — Additional file 4: Figure S2. Mean kinship of the estimated current population of 31,832 Leonberger dogs per country. The proportion of dogs belonging to the three groups that indicate the increasing relatedness to the whole population, is shown as green (MK < 0.31), yellow (MK = [0.31–0.32]), and orange (MK > 0.32) by country. The total number of dogs recorded in each country is shown in red at the top of the corresponding columns. [file 12711_2020_581_MOESM4_ESM.tif]

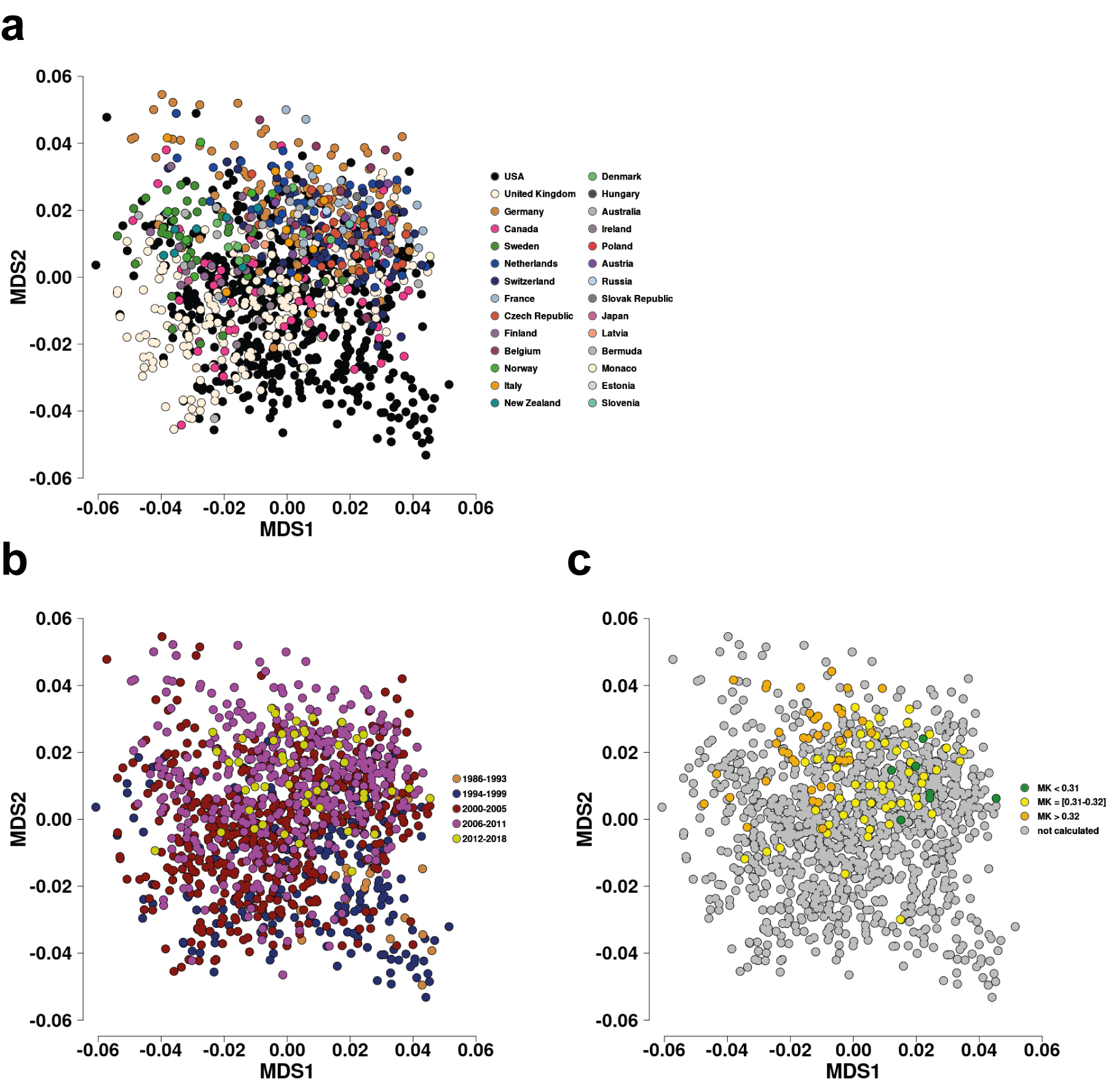

Supplement: Supplementary file 6 — Additional file 6: Figure S3. MDS plots of the 1203 Leonberger dogs highlighted by different groups. Description: Panel (a) shows the distribution of the dogs by country. Panel (b) shows the dogs divided into five groups by their year of birth. This is also reflected in panel (c), where the dogs are coded by their calculated MK coefficient, which was not determined for the older dogs. [file 12711_2020_581_MOESM6_ESM.tif]
