# Supplementary material for: Genomic diversity and population structure of the Leonberger dog breed
Source: Genet Sel Evol. 2020 Oct 14;52:61. doi: 10.1186/s12711-020-00581-3 (PMC7557023; doi:10.1186/s12711-020-00581-3)
Supplement: Supplementary file 7 — Additional file 7: Figure S4. Proportion of dogs with a SNP within a ROH on each of the 38 autosomes. Individual Manhattan plots showing the percentage of 1203 dogs genotyped on the SNP array that share a SNP within a ROH for each canine autosome. [file 12711_2020_581_MOESM7_ESM.pdf]

Additional file 7 Figure S4. Proportion of dogs with a SNP within a ROH on each of the 38 autosomes.

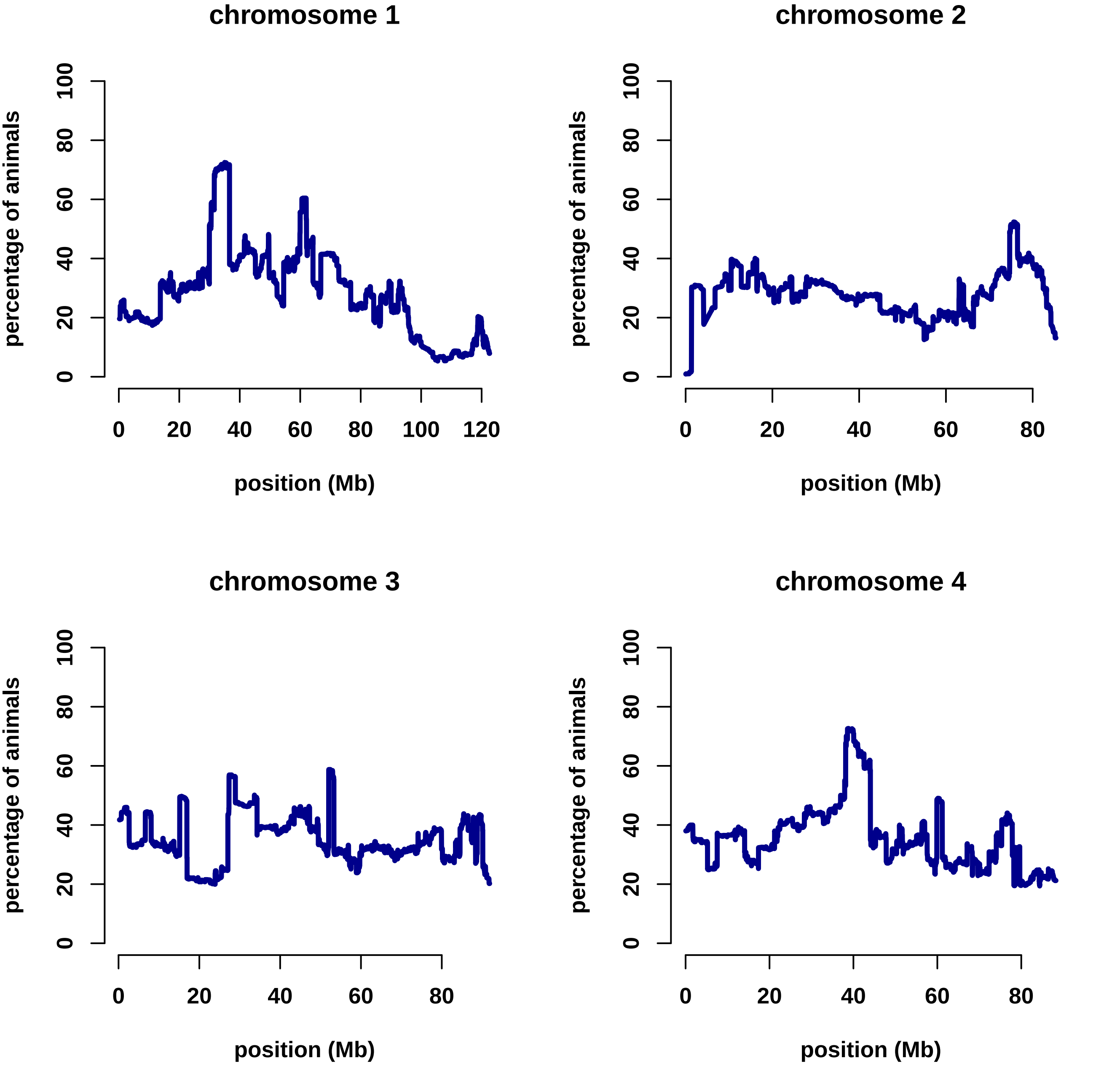

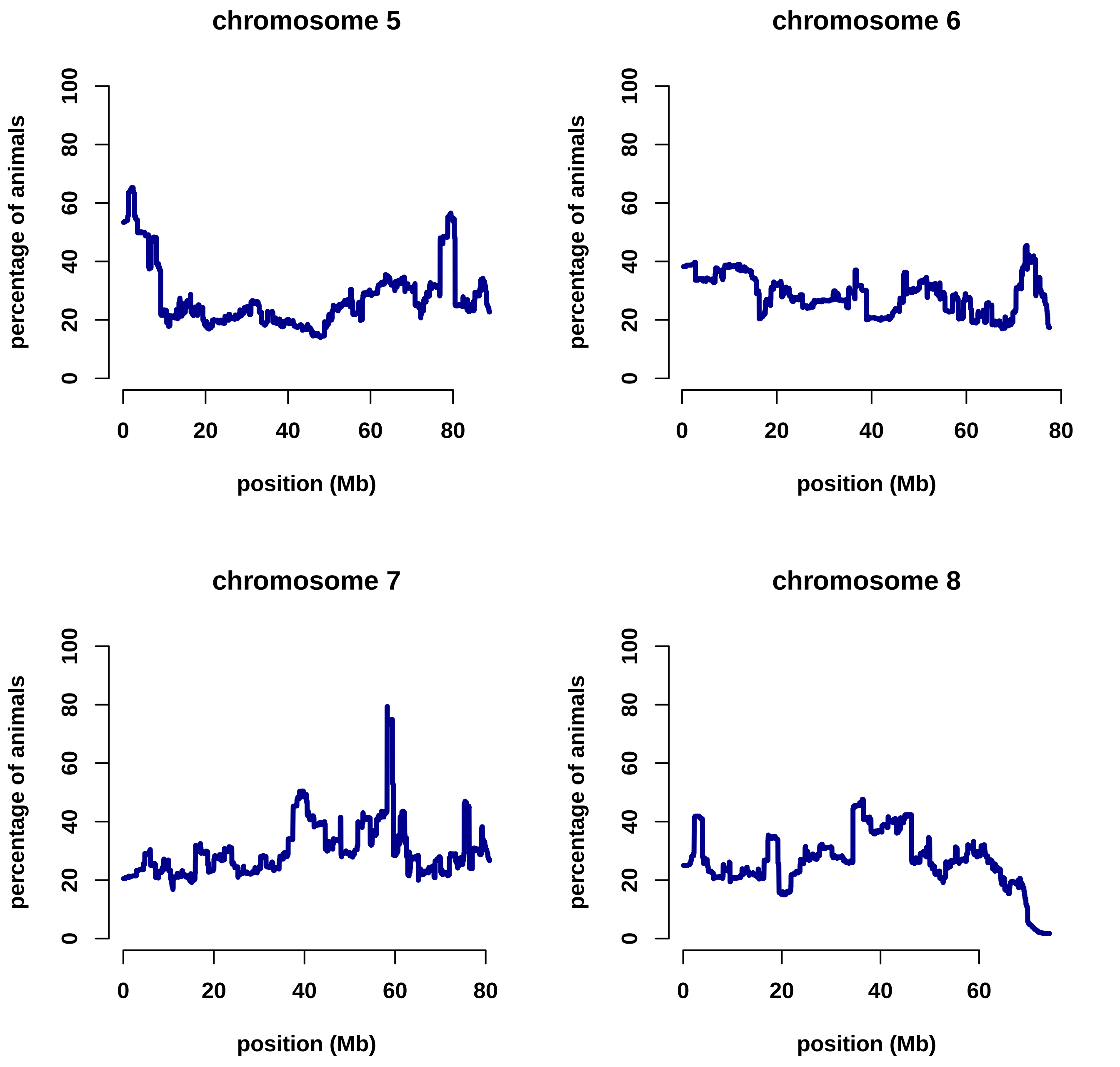
chromosome 5
chromosome 6
chromosome 7
chromosome 8
percentage of animals
position (Mb)
0
20
40
60
80
100

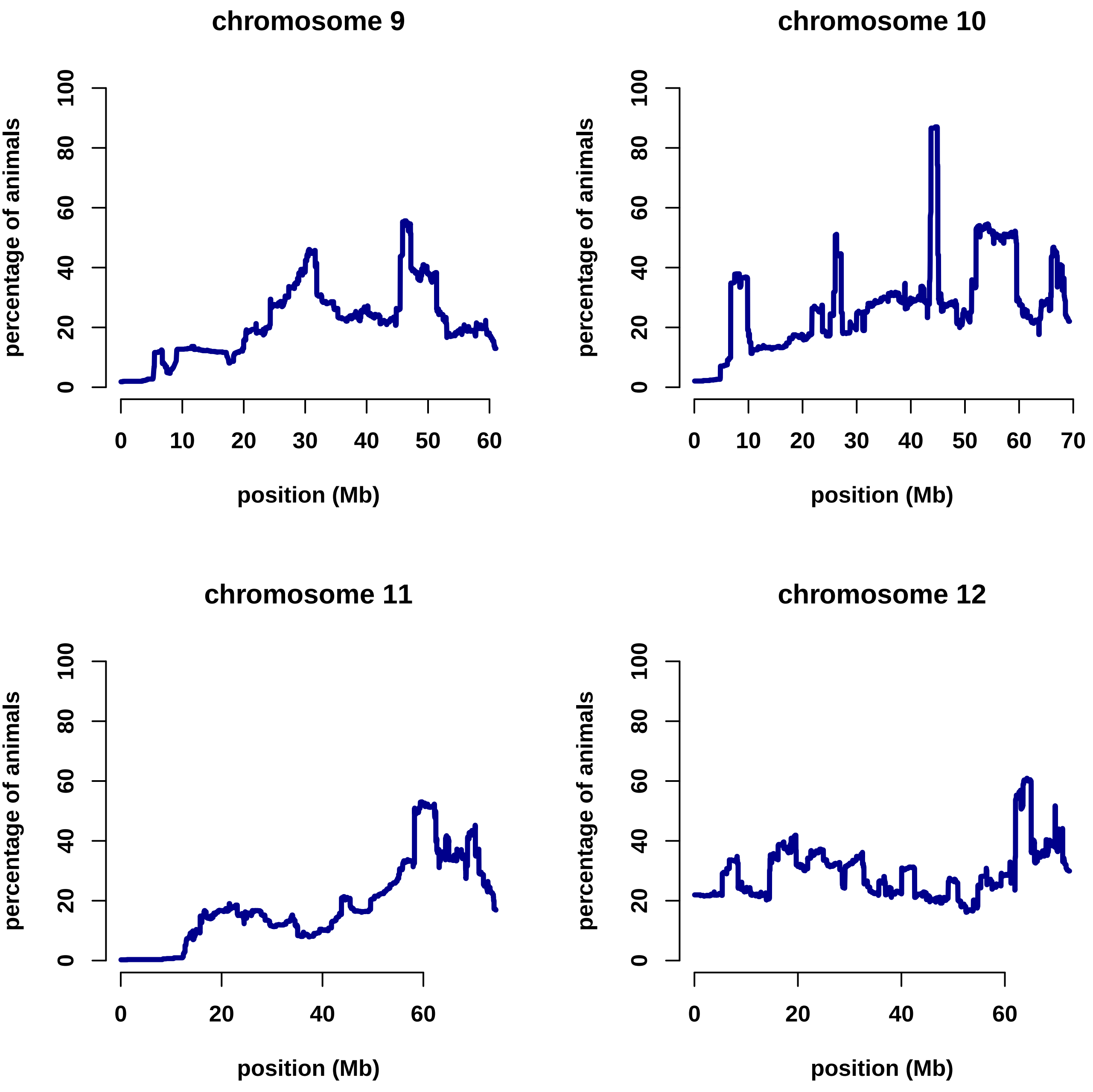

chromosome 9
chromosome 10
chromosome 11
chromosome 12
percentage of animals
position (Mb)
0
20
40
60
80
100
0
10
20
30
40
50
60
70

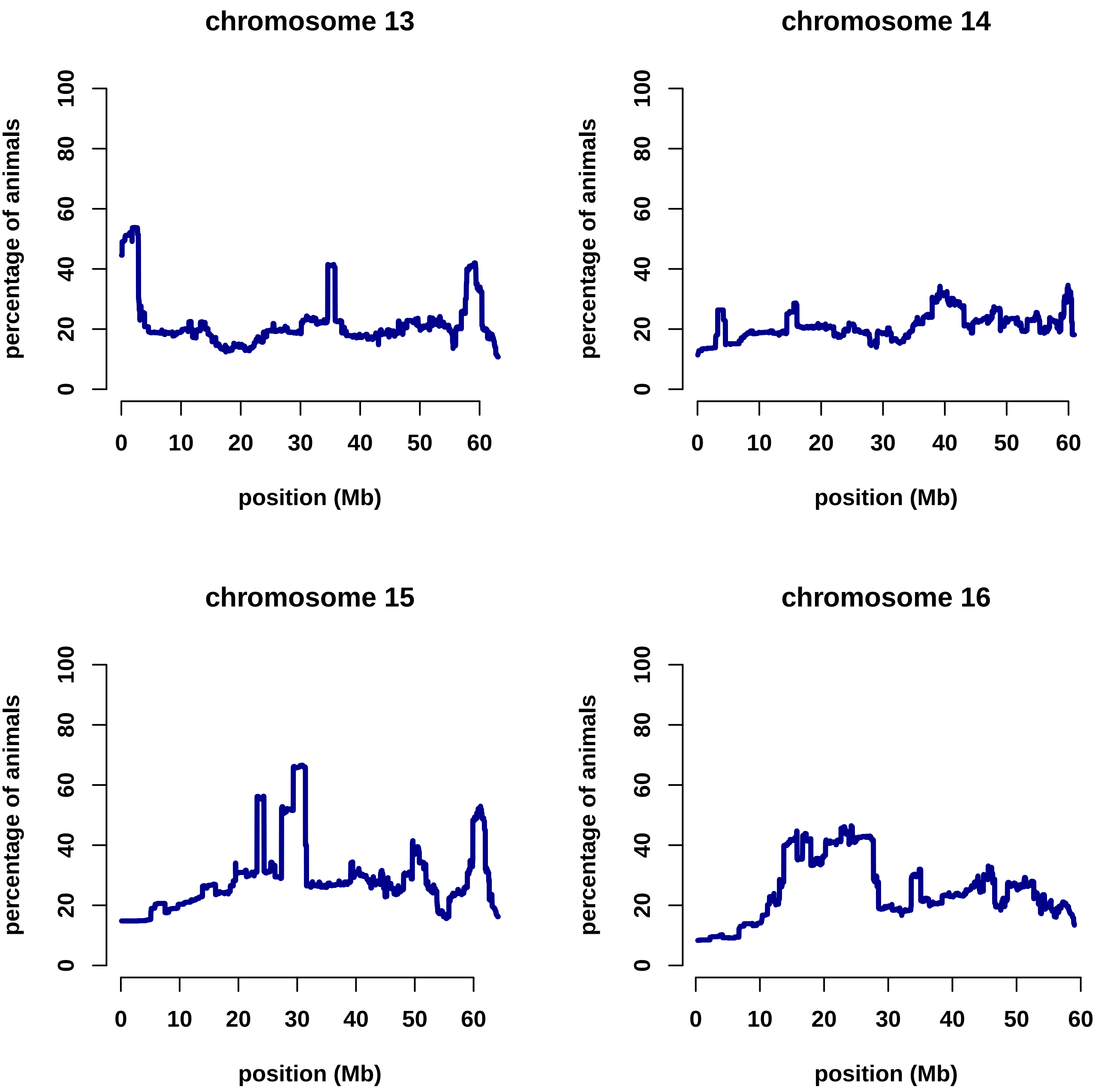
chromosome 13
chromosome 14
chromosome 15
chromosome 16
percentage of animals
position (Mb)
0
20
40
60
80
100
0
10
20
30
40
50
60

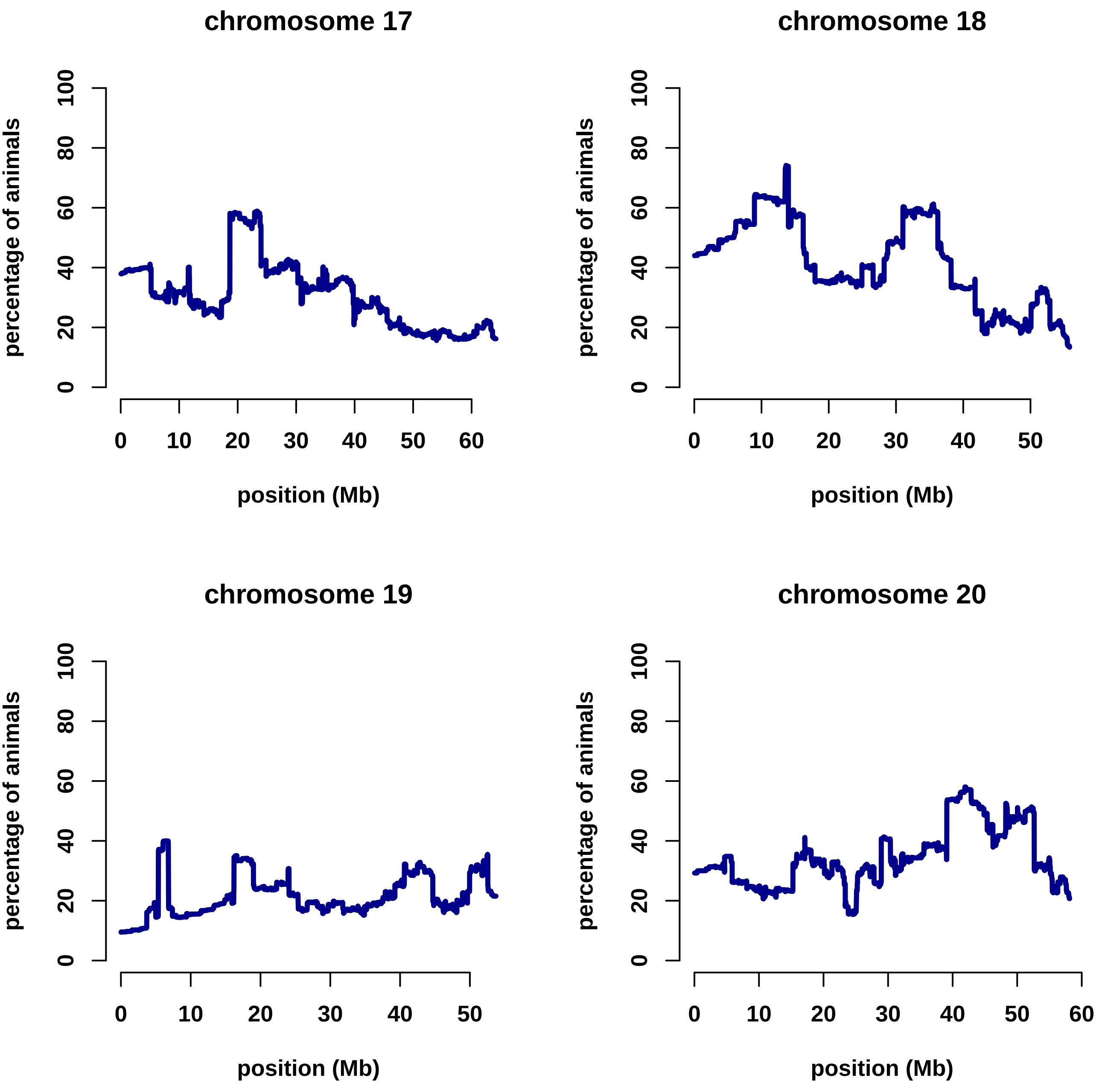

chromosome 17
chromosome 18
chromosome 19
chromosome 20
percentage of animals
position (Mb)
0
20
40
60
80
100
0
10
20
30
40
50
60

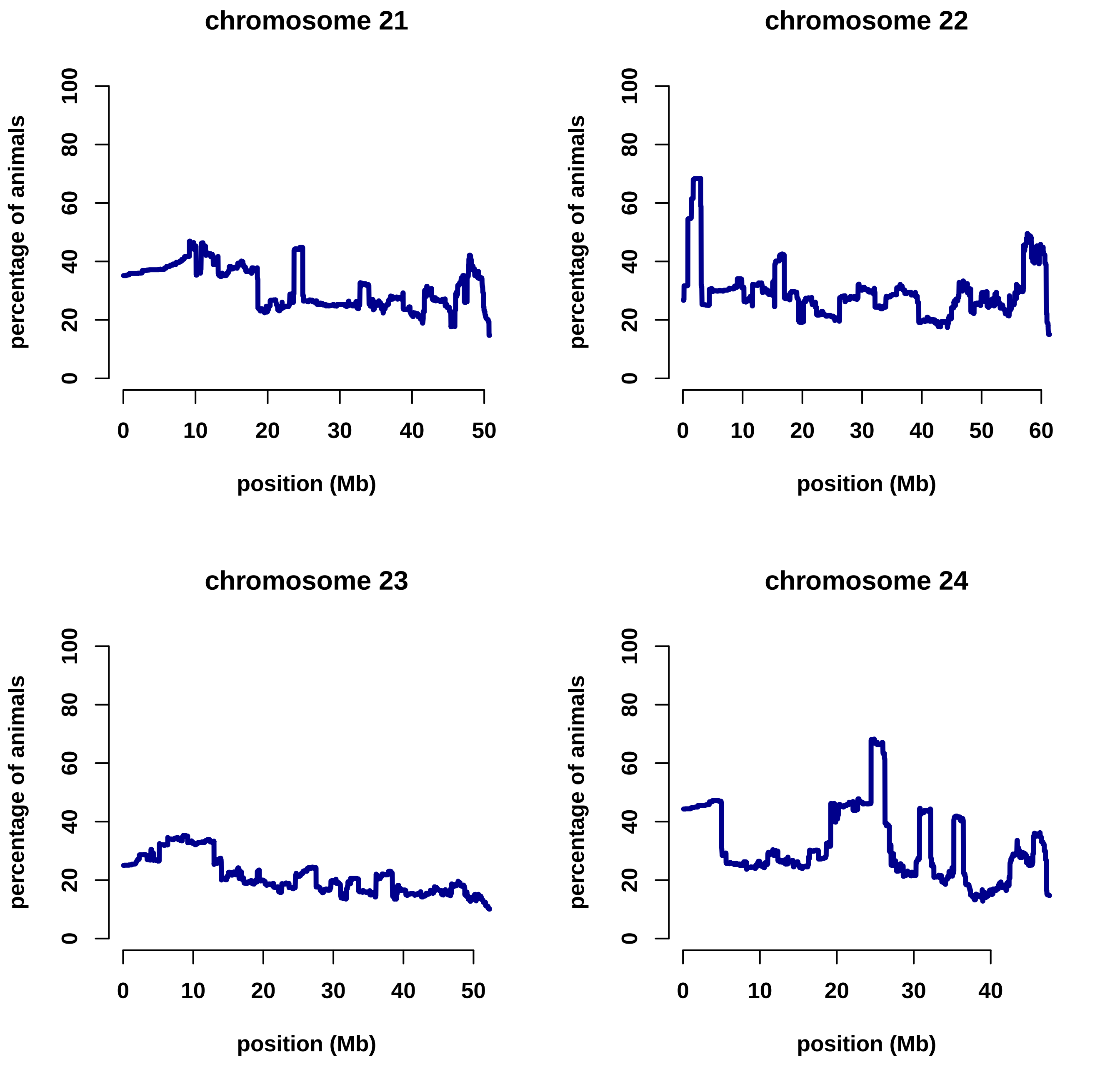
chromosome 21
chromosome 22
chromosome 23
chromosome 24
percentage of animals
position (Mb)
0
20
40
60
80
100
0
10
20
30
40
50
60

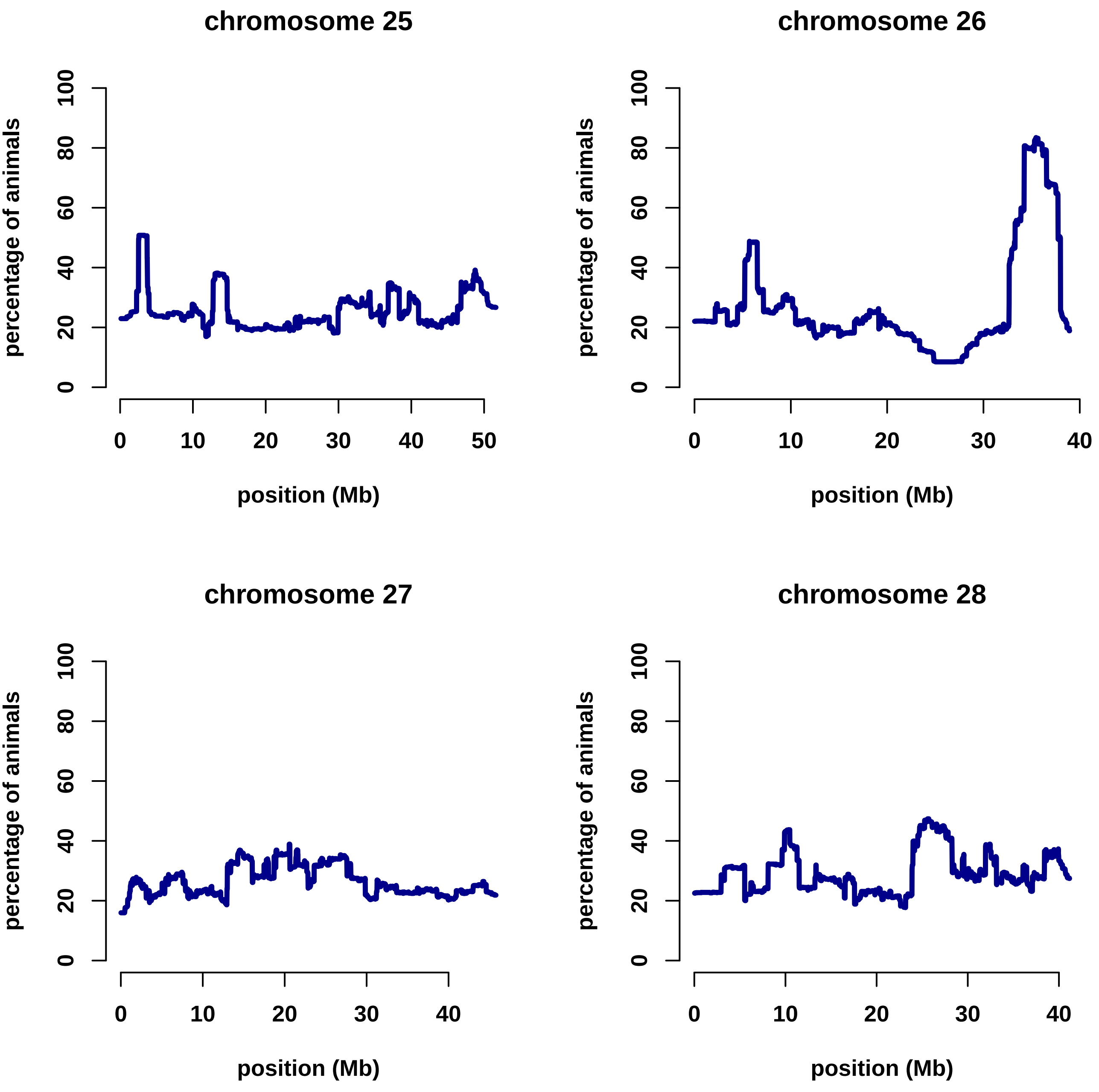

chromosome 25
chromosome 26
chromosome 27
chromosome 28
percentage of animals
position (Mb)
0
20
40
60
80
100
10
30
50

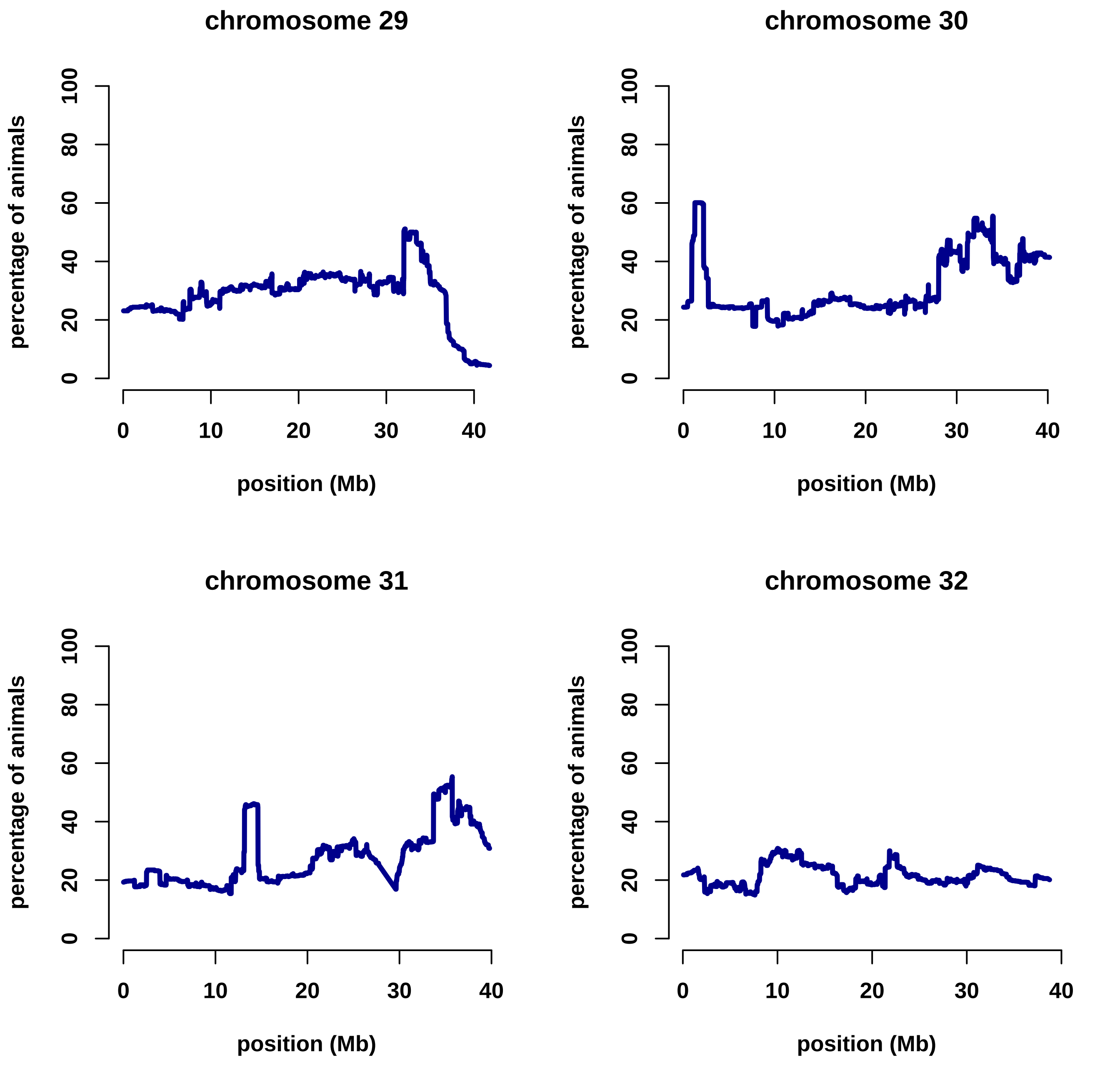

chromosome 29
chromosome 30
chromosome 31
chromosome 32
percentage of animals
position (Mb)
0
20
40
60
80
100
0
10
20
30
40

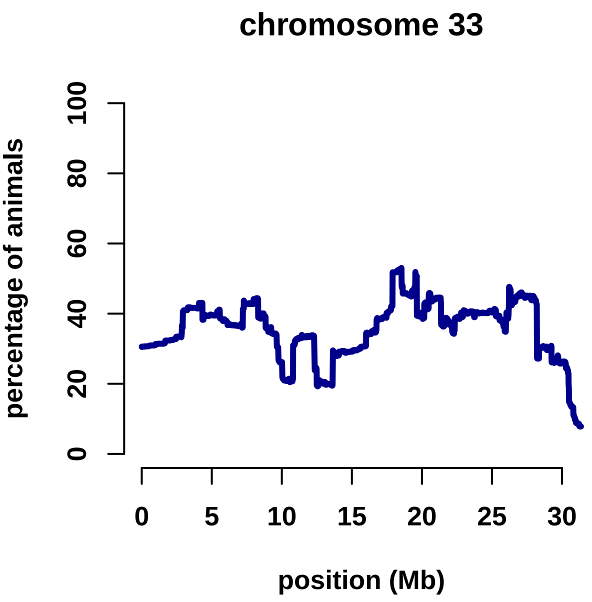
chromosome 33
percentage of animals
0
20
40
60
80
100
0
5
10
15
20
25
30
position (Mb)

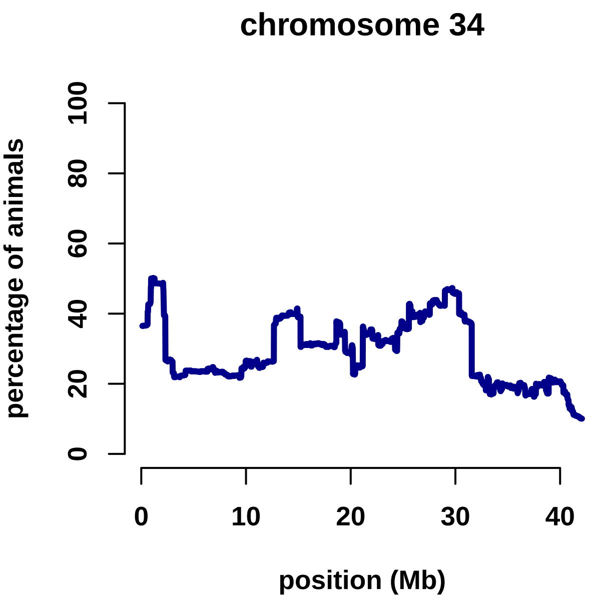
chromosome 34
percentage of animals
0
20
40
60
80
100
0
10
20
30
40
position (Mb)

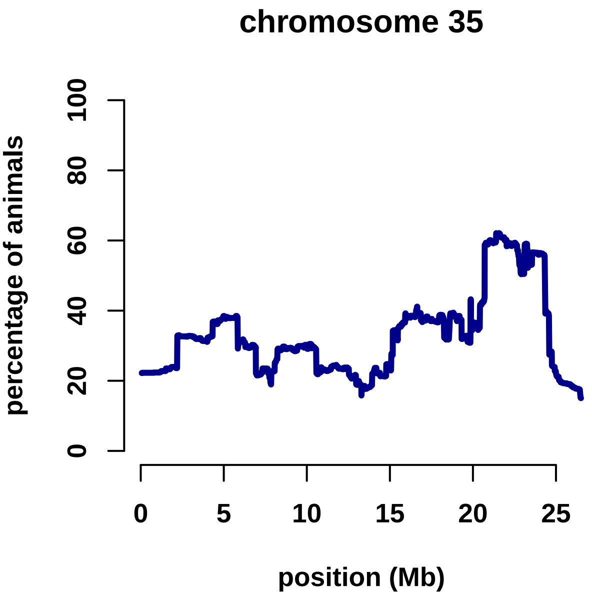
chromosome 35
percentage of animals
0
20
40
60
80
100
0
5
10
15
20
25
position (Mb)

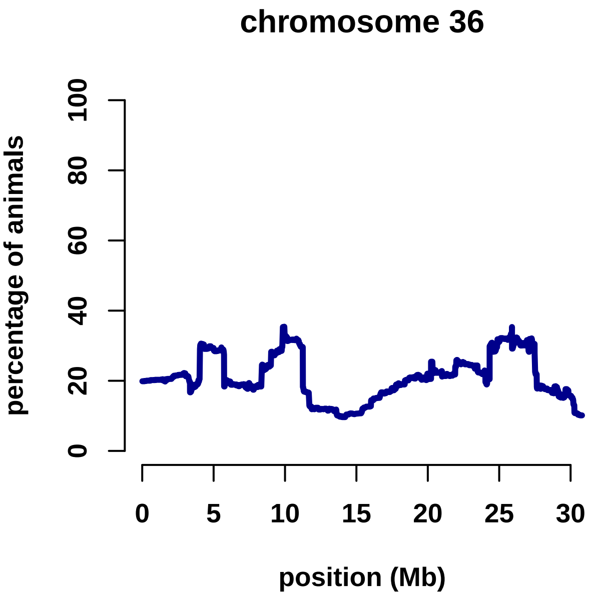
chromosome 36
percentage of animals
0
20
40
60
80
100
0
5
10
15
20
25
30
position (Mb)

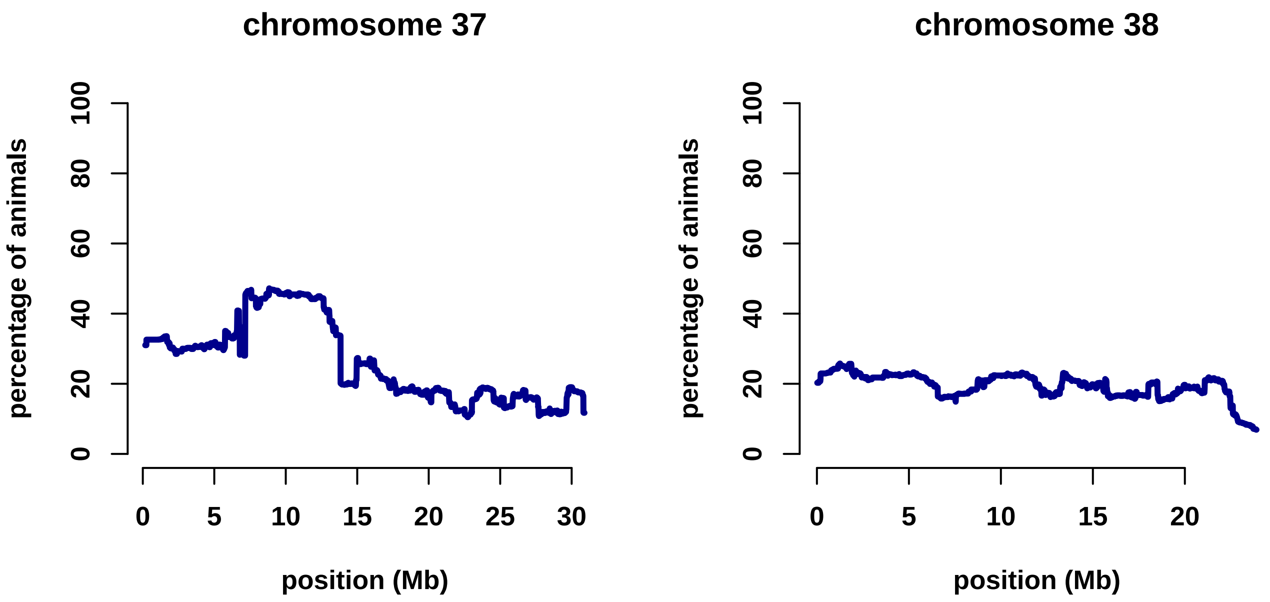
chromosome 37
chromosome 38
percentage of animals
0
20
40
60
80
100
0
5
10
15
20
25
30
position (Mb)
